# Assessment of Knowledge, Attitude, and Practice (KAP) Among Nurses on Oral Care for Intensive Care Unit Patients in Riyadh, Saudi Arabia: A Cross-Sectional Study

**DOI:** 10.7759/cureus.50682

**Published:** 2023-12-17

**Authors:** Kiran Iyer, Khalifah AlKhalifah, Bader Nashir Alshahrani, Saed Saeed Ibrahim Alghamdi, Sultan Albishi, Abdulrahman Abdulelah A Alsheraihi, Nawaf Al Sudairy

**Affiliations:** 1 Dental Public Health, King Saud Bin Abdulaziz University for Health Sciences, King Abdullah International Medical Research Centre, Ministry of National Guard Health Affairs, Riyadh, SAU; 2 College of Dentistry, King Saud Bin Abdulaziz University for Health Sciences, King Abdullah International Medical Research Centre, Ministry of National Guard Health Affairs, Riyadh, SAU; 3 College of Dentistry, King Saud Bin Abdulaziz University for Health Sciences, Riyadh, SAU

**Keywords:** intensive care units, oral health, practice, attitude, knowledge, nursing

## Abstract

Background: Oral care of intensive care unit (ICU) bound individuals is essential for overall health outcomes and to prevent complications. Nurses, who are the primary caregivers, should possess adequate knowledge, attitude, and practice (KAP) in this regard to provide optimal care to these patients. There are no standardized guidelines existing at present in this regard, making the practice of oral care more challenging. There is a diversified representation of nurses who practice in this region of the world and have not been analyzed in the past. This study would like to address this paucity of data. Hence, the aim of the present study was to evaluate the knowledge, attitude, and practice of nurses regarding oral health care in ICU patients along with analyzing any existing hospital-based policies related to oral care.

Materials and methods: A cross-sectional study was conducted among 230 nurses practicing at ICU of the National Guard Health Affairs (NGHAs) Hospital, King Abdulaziz Medical City, in Riyadh City, Saudi Arabia. Nurses responded to 22 closed-ended questionnaires, which were adopted after content validation and reliability assessment. Descriptive statistics, chi-square analysis, and multinomial logistic regression were carried out using the Statistical Package for the Social Sciences (SPSS, Version 20, 2011; IBM Corp., Armonk, USA).

Results: A total of 230 (51.1%) nurses responded. The mean working experience of 12.6 (±7.5) years and ICU experience of 10.6 (±6.7) years were observed. Seventy-four (32.2%) nurses mentioned they received oral care training for ICU patients as part of their degree. A significant variation (p=0.03) in response was observed based on qualification concerning the knowledge of nurses if improper oral care among ICU patients could cause systemic complications. Nurses with less than five years’ experience were more likely to provide oral care only once per day compared to nurses with higher experience (>10 years) (OR: 2.97, p=0.00, 95% CI: 2.40-12.2).

Conclusion: There were certain knowledge, attitude, and practice-based questions that elicited significant differences in responses based on the qualifications and experience of the nurses. Overall, the nurses did possess fair knowledge and favorable attitudes towards oral care in these patients.

## Introduction

Oral health is made up of complex microbial flora, and the oral cavity acts as a reservoir of these microorganisms [1]. Critically ill patients in the intensive care unit (ICU) suffer from poor oral health due to morbidity, decreased dexterity, and variable conscious state [2]. Surgeons frequently have encountered issues regarding oral hygiene and oral health care in three broad settings: patients unable to consume oral fluids or diet; ventilated patients in the intensive care unit; or those requiring palliative assistance. However, many patients post-surgery have poor oral hygiene accentuated by xerostomia, chemotherapy, and dehydration [3].

The Centers for Disease Control and Prevention guidelines report that microorganisms enter the lungs through the oropharyngeal region, during the process of microaspiration [4]. Additionally, when a ventilator is used in these patients, it leads to the pooling of discharge and colonization by opportunistic bacteria in the posterior region of the oropharynx and larynx, which, on aspiration or microaspiration into the lungs, leads to hospital-based pneumonia or ventilator-acquired pneumonia (VAP) [5]. This type of infection amounts to 33% of all hospital-based infections in ICU [6]. Studies in the past have established bacterial colonization in the oropharyngeal region leading to pneumonia in patient's dependent on mechanical ventilation [5,7,8]. Furthermore, the pathogenic microorganisms of the oral cavity form an important etiological factor of upper respiratory tract infections in these patients; therefore, oral care becomes a high priority in health-care delivery, a factor that can avert complications among these patients.

Further oral assessment and care form a prerequisite, as most patients in ICUs are on drugs that have side effects such as reduced salivary flow (xerostomia), which leads to the colonization of the oral cavity with opportunistic infections such as *Candida albicans*. Also, oropharyngeal morbidity in patients causes pain, and alteration in deglutination that can lead to difficulty in maintaining an adequate dietary intake [6].

Though nurses often have their own approaches to providing patient care, routine work varies based on the nurse's education, experience, and career background in nursing care [5]. Studies have observed that the provision of oral hygiene is allocated a low priority in several nursing undergraduate programs, and although considered to be a basic nursing practice, it is at risk of assuming a lesser priority when rendering care to intensive care patients. This shortfall in the prioritization of oral hygiene, despite incriminating evidence existing in literature for an association between poor oral health and VAP, may be due to a lack of knowledge or appreciation of its importance by registered nurses [9,10]. 

Nursing care for ICU patients at hospitals in Riyadh City, Kingdom of Saudi Arabia (KSA), is carried out by qualified nurses who hail from diverse educational backgrounds and have experience owing to their country of origin. Therefore, analyzing the knowledge, attitude, and practice regarding oral care practice by nurses posted for patient care at ICUs in various departments becomes of foremost importance. On a detailed assessment of the literature, no studies were carried out or reported from this region, contributing to this topic. Hence, this study was undertaken to evaluate the knowledge, attitude, and practice of nurses regarding oral health care in ICU patients, along with analyzing any existing hospital-based policies related to oral care being implemented.

## Materials and methods

A descriptive, cross-sectional study was conducted to assess the KAP among nurses in oral care for ICU patients. Before data collection, requisite Institutional Review Board (IRB) approval (IRB/1690/22) from King Abdullah International Medical Research Center (KAIMRC) for the study protocol was obtained; permission from the concerned authorities in the nursing services research committee (RM/2023/01/15) was also obtained before the commencement of the study at the National Guard Health Affairs (NGHA), Riyadh City; and informed consent from all nurses willing to be enrolled was collected.

Sampling, sample frame, and sample size

Data collection was completed over one and half months (March 2023-May 2023), and study participants (nurses) were recruited based on a non-probability purposive sampling technique from the hospitals (including the specialized hospitals for cardiovascular and pediatric patients) of the National Guard Health Affairs (NGHA) at Riyadh City, King Abdulaziz Medical City, Saudi Arabia. Nurses providing informed consent and having experience in providing patient care at the ICU were included in the study. Nurses who were not willing to participate in the study and did not provide informed consent and those without any experience of ICU postings were excluded from the study. The sample size was estimated at 220 using G Power software (3.1.9.4) (Heinrich-Heine-Universität Düsseldorf, Düsseldorf, Germany), with a confidence interval (CI) of 95%, power of study set at 80%, and considering the nurse-to-population ratio (4.5 per 1000) as per data of Saudi Arabia [11].

Questionnaire

Data was collected using a self-reported questionnaire, which was administered to the nurses through emails and Google Forms via QR codes installed at nursing stations and outside ICUs. Data was collected over one and a half months and finally analyzed for incomplete forms and duplicates, if any. Before data collection, the questionnaire was assessed for content validity by six experts using an ordinal scale (1-not relevant, 2-somewhat relevant, 3-quite relevant, and 4-highly relevant) as per Lynn MR (1986), and the item-level content validity index (0.85) and scale-level content validity index (0.95) were measured [12]. The reliability of the questionnaire was assessed by test-retest on a pilot sample of 20 nurses (10%) at a two-week interval, and a Cronbach’s α (0.86) value was ascertained for the same.

The questionnaire consisted of three sections (knowledge, attitude, and practice) with 22 questions, apart from the demographic details. The demographic details form the exploratory variables; the variables assessed under KAP were the dependent variables, assessed as against the exploratory variables. The knowledge component was assessed using six questions with choices (yes/no/not sure). The attitude component consists of eight items with choices on a Likert scale ranging from 1 (strongly disagree) to 5 (strongly agree), and the practice component, consisting of seven items, was assessed using a combination of choices such as (yes/no/not sure) and (select all that apply). The questionnaire has been adopted from two studies after modification [13,14].

Statistical analysis

The data was entered in Microsoft Excel (Microsoft Corp., New York, USA) and transferred to IBM SPSS statistical software (Windows Version 20, 2011; IBM Corp., Armonk, USA) for analysis. Descriptive statistics was used to describe all the qualitative variables and demographic data. Pearson's chi-square analysis was used to find significance in KAP with exploratory variables. Significant variables were explored further with multinomial logistic regression analysis.

## Results

Demographics

A total of 450 nurses posted at various ICUs were approached with the self-administered questionnaire; all correspondence was carried out through the nurse station manager at each of the ICUs. After three consecutive follow-ups at an interval of 15 days each, we managed to get a total of 230 (51.1%) responses. The mean age of the nurses in the study was found to be 36.2 (±8.0). Of the 230 nurses who responded, 42 (18.3%) were male and 188 (81.7%) were female, respectively. Among the nurses, a mean working experience of 12.6 (±7.5) years and ICU experience of 10.6 (±6.7) years were observed. An interesting fact observed in this study is the diverse work culture where nurses represent about 15 countries, with about 89 (38.7%) nurses from the Philippines being the highest (Table 1).

**Table 1:** Mean, frequency, and percentage of independent demographic variables in the study. N: total Count, n: frequency.

Demographic variables	N	Mean years	Std. deviation (±)	Frequency (n)	Percentage (%)
Age in years	230	36.29	8.03	-	-
Gender	230	-	-	Male (42)	18.3
				Female (188)	81.7
Total work experience in nursing care	230	12.69	7.55	-	-
Total work experience in ICU	230	10.66	6.76	-	-
Country: Philippines				89	38.7
Malaysia	-	-	-	76	33.0
South Korea	-	-	-	9	3.9
India	-	-	-	10	4.3
Saudi Arabia	-	-	-	26	11.3
Canada	-	-	-	2	0.9
Egypt	-	-	-	1	0.4
Czech Republic	-	-	-	4	1.7
South Africa	-	-	-	5	2.2
Lebanon	-	-	-	1	0.4
United Kingdom	-	-	-	2	0.9
Slovenia	-	-	-	1	0.4
Portugal	-	-	-	1	0.4
Spain	-	-	-	1	0.4
Serbia	-	-	-	2	0.9
Total				230	100
Specialty: cardiovascular				65	28.3
Respiratory				24	10.4
ICU (general)				98	42.6
Surgical	-	-	-	10	4.3
Pediatric				20	8.7
Trauma				9	3.9
Other				4	1.7
Total				230	100

Knowledge, attitude, and practice

Chi-square analysis of knowledge, attitude, and practice (dependent variables) with the type of nursing qualification (master's degree, bachelor's degree, and diploma), years of working at ICU, and working at ICU specialty type (independent variables), a significant variation (p=0.03) in response was observed based on qualification concerning knowledge on improper oral care among ICU patients could cause systemic complications; similarly, there was a significant (p=0.01) variation in response for this question when analyzed based on years of ICU experience among nurses (Table 2).

**Table 2:** Knowledge and attitude responses based on nursing qualification, country of origin, and years of experience related to oral care for intensive care unit patients. n: frequency, %: percentage, chi-square analysis, *P<0.05.

Question	Variable	n (%)					Pearson chi-square value
Does improper or insufficient oral care in ICU patients cause systemic complications?	Nursing qualification	Yes		No	Not sure		0.03*
	Master’s degree	12 (100%)		0 (0%)	0 (0%)		
	Bachelor’s degree	149 (90.3%)		9 (5.5%)	7 (4.2%)		
	Diploma	37 (69.8%)		8 (15.1%)	8 (15.1%)		
	Years of experience	Yes		No	Not sure		0.01*
	<5 Years	55 (96.5%)		0 (0%)	2 (3.5%)		
	6-10 Years	59 (76.6%)		10 (13.0%)	8 (10.4%)		
	>10 Years	84 (87.5%)		7 (7.3%)	5 (5.2%)		
Can ICU patients develop oral complications due to their medication?	Years of experience	Yes		No	Not sure		0.20
	<5 Years	48 (84.2%)		4 (7.0%)	5 (8.8%)		
	6-10 Years	64 (83.1%)		4 (5.2%)	9 (11.7%)		
	>10 Years	87 (93.5%)		3 (3.2%)	3 (3.2%)		
Does poor oral hygiene cause ventilator-associated pneumonia (VAP) in ICU patients who are on ventilators?	Nursing qualification	Yes		No	Not sure		0.01*
	Master’s degree	11 (91.7%)		1 (8.3%)	0 (0%)		
	Bachelor’s degree	156 (94.5%)		5 (3.0%)	4 (2.4%)		
	Diploma	45 (84.9%)		1 (1.9%)	7 (13.2%)		
Have you ever felt cleaning the oral cavity of ICU patients is a very unpleasant task?	Nursing qualification	Strongly disagree	Disagree	Neither disagree/agree	Agree	Strongly agree	0.02*
	Master’s degree	4 (33.3%)	3 (25.0%)	0 (0%)	5 (41.7%)	0 (0%)	
	Bachelor’s degree	43 (26.4%)	70 (42.9%)	32 (19.6%)	12 (7.4%)	6 (3.7%)	
	Diploma	13 (21.7%)	24 (24.7%)	8 (20.0%)	7 (29.2%)	1 (14.3%)	
Do you find it difficult to clean the oral cavity of ICU patients?	Nursing qualification	Strongly disagree	Disagree	Neither disagree/agree	Agree	Strongly agree	0.01*
	Master’s degree	3 (25%)	1 (8.3%)	5 (41.7%)	2 (16.1%)	1 (8.3%)	
	Bachelor’s degree	18 (10.9%)	82 (49.7%)	28 (17.0%)	34 (20.6%)	3 (1.8%)	
	Diploma	4 (7.5%)	20 (37.7%)	12 (22.6%)	12 (22.6%)	5 (9.4%)	
Do you feel it is the nurse’s responsibility for the provision of oral care in your unit?	Country	Strongly disagree	Disagree	Neither disagree/agree	Agree	Strongly agree	0.00*
	Philippines	8 (9.1%)	0 (0.0%)	0 (0.0%)	34 (38.6%)	46 (52.3%)	
	Malaysia	1 (1.3%)	0 (0.0%)	1 (1.3%)	32 (42.1%)	42 (55.3%)	
	Saudi-Arabia	2 (8.0%)	0 (0.0%)	5 (20.0%)	8 (32.0%)	10 (40.0%)	
Do you feel the mouth of most ventilated patients get worse no matter what oral care you give?	Country	Strongly Disagree	Disagree	Neither Disagree/Agree	Agree	Strongly Agree	0.04*
	Philippines	5 (5.7%)	36 (40.9%)	28 (31.8%)	18 (20.5%)	1 (1.1%)	
	Malaysia	4 (5.3%)	29 (38.2%)	20 (26.3%)	17 (22.4%)	6 (7.9%)	
	Saudi Arabia	4 (16.0%)	13 (52.0%)	4 (16.0%)	2 (8.0%)	2 (8.0%)	
Do you think that each patient admitted to ICU should be reviewed by a dentist to assess oral care needs and provide a management plan?	Country	Strongly disagree	Disagree	Neither disagree/agree	Agree	Strongly agree	0.00*
	Philippines	1 (1.1%)	9 (10.2%)	31 (35.2%)	38 (43.2%)	9 (10.2%)	
	Malaysia	3 (3.9%)	12 (15.8%)	38 (50.0%)	18 (23.7%)	5 (6.6%)	
	Saudi Arabia	1 (4.0%)	4 (16%)	7 (28%)	10 (40%)	3 (12%)	

Nurses, irrespective of their experience, responded equivocally (non-significant, p=0.20) to the knowledge-based question, "Can ICU patients develop oral complications due to their medication?". Interestingly, there was a significant variation (p=0.01) in response to "Does poor oral hygiene cause Ventilator-Associated Pneumonia (VAP) in ICU patients who are on a ventilator?,” with a higher percentage of bachelor's degree and diploma holders responding as not sure for the same compared to their counterparts who had a master's degree (Table 2).

Attitude-based questions were observed against the nursing qualifications and nationality of the nurse. Questions such as “Have you ever felt cleaning the oral cavity of ICU patients is a very unpleasant task?” and “Do you find it difficult to clean the oral cavity of ICU patients?” when observed against the type of nursing qualification elicited a significant variation (p=0.02 and p=0.01, respectively) in response. A higher percentage of those with bachelor's degrees and diplomas seemed to agree with difficulty in accessing and cleaning the oral cavity. When attitudinal variation based on the nationality of nurses was studied, a significant (p=0.00, p=0.04, and p=0.00) relation in the variation of response was observed for the following questions: “Do you feel its nurse’s responsibility for the provision of oral care in your unit?", "Do you feel the mouth of most ventilated patients get worse no matter what oral care you give?” and “Do you think that each patient admitted to ICU should be reviewed by a dentist to assess oral care needs and provide a management plan?”. Responses of nurses from the Philippines and Malaysia (the predominant population of nurses) were analyzed along with the regional (Saudi-Arabian nurse) to observe variation in response. Though other nationalities were analyzed for responses, only these have been presented in the study owing to the diverse nationalities among the study population (Table 2).

Practice-oriented questions were analyzed for frequency and against the nurse's qualification and years of experience; most of them, 186 (80.9%), responded that they do provide three or more times oral care for ICU patients each day. About 101 (43.9%) of the nurses mentioned they spend about two to five minutes assessing and delivering oral care to ICU patients at each sitting. Just about half of the respondents, 132 (57.4%), confirmed having a formal protocol or policy to assess or provide care for the oral cavity at their ICU. Similarly, a majority of 201 (87.4%) of the respondents affirmatively responded to documenting the oral cavity findings of these patients. About 143 (62.1%) of nurses confirmed the use of adult toothbrush and paste, whereas only 117 (50.8%) of the respondents mentioned that pediatric toothbrush was used where required, 194 (84.3%) of them mentioned that foam swabs were used routinely to maintain oral hygiene in these patients and about 136 (59.1%) reported using chlorhexidine as mouthwash among these patients. None of the nurses responded to the use of artificial saliva or antibiotic gels in routine care.

Interestingly, nurses with bachelor's and diploma degrees significantly (p=0.04) responded to providing oral care more frequently (three or more times) than their counterparts who had a master's degree. When the duration of assessment and care of the oral cavity was considered, those with bachelor's and diplomas were significantly (p=0.00) more likely to spend less than two minutes at each sitting with the patient.

On multinomial logistic regression analysis to assess the significance of attitude and practice with years of ICU experience, it was observed that those with less than five years of experience (OR: -2.91, p=0.027, 95% CI: 0.004-0.71) were significantly less likely to agree with a need of a dentist to assess oral care needs and provide a management plan for patients admitted to the ICU. Interestingly, the same group of nurses were more likely to provide oral care only once per day compared to nurses with higher experience (OR: 2.97, p=0.00, 95% CI: 2.40-12.2) (Table 3).

**Table 3:** Multinomial regression to assess the significance of attitude and practice with years of ICU experience. B: coefficients, df: degrees of freedom, *P<0.05, Exp (B): odds ratio.

Question	Response	Years of experience	B	df	Sig.	Exp (B)	95% Confidence interval for Exp (B)	
							Lower bound	Upper bound
Do you think that each patient admitted to ICU should be reviewed by a dentist to assess oral care needs and provide a management plan?	Strongly agree	(<5 Years experience)	-2.91	1	0.02*	0.05	0.00	0.71
		(6-10 Years experience)	-1.8	1	0.07	0.15	0.02	1.16
Do you feel it is the nurse’s responsibility for the provision of oral care in your unit?	Strongly agree	(<5 Years experience)	-1.0	1	0.19	0.34	0.07	1.68
		(6-10 Years experience)	-1.3	1	0.09	0.26	0.05	1.25
How frequently do you provide oral care to ICU patients each in your unit?	Once per day	(<5 Years experience)	2.97	1	0.00*	19.61	2.40	12.20
		(6-10 Years experience)	0.31	1	0.82	1.37	0.08	22.34
At your current ICU unit is there a formal protocol for the assessment of the oral cavity/oral health needs of patients?	Not sure	(<5 Years experience)	1.35	1	0.00*	3.86	1.55	9.14
		(6-10 Years experience)	0.07	1	0.11	2.09	0.83	5.29

## Discussion

Literature does suggest that there exists no standard published protocol for oral care of patients admitted at the ICU, making it even more essential to analyze the knowledge, attitude, and practice of nurses toward oral care among these patients. This would help observe and report regional variations and emphasize the development of policy and protocol to standardize the quality of oral care delivery in these patients.

This study observed a very diverse group of nurses from various nationalities (15 countries) practicing in large multiple specialty hospitals in one medical city (King Abdulaziz Medical City). This observation of diversity and its reporting have not been carried out in any of the previous studies.

Though only about 152 (66%) of the nurses reported having received some form of training in the assessment and provision of oral care in ICU-bound patients, a high percentage of nurses in our study had the appropriate knowledge about oral complications that may develop due to medication and exposure to ventilation-associated pneumonia (VAP) among these patients. Findings about knowledge among nurses were contrary to previous studies by Haghighat et al. and Khojastehfar et al. [15] and Chan et al. [16] who reported low to moderate levels of knowledge in their population of nurses being surveyed. Our findings on knowledge levels are found to be in line with studies by Philip et al. [14] and Gaffar et al. [17].

An interesting observation in our study was that a high percentage of nurses did mention that they knew 195 (84.8%) oral care for ICU patients with a denture. This contrasts with studies by Pettit et al. [18] and Philip et al. [14], who observed a lack of awareness of this aspect among the nurses surveyed as part of their study. Denture care and oral care among denture-wearing ICU patients form a significant part of overall care, as improper care leads to complications such as aspirational pneumonia, as reported by Haresaku et al. [19].

This study assessed for variation in knowledge based on qualification and years of experience; similarly, we tried to assess if there existed variation in the attitude of nurses towards oral care among ICU patients based on the qualification and nationality of these nurses. Years of experience was considered for assessment as nurses also learn oral care provision for these patients informally on the job, as confirmed by most studies.

In the present study, 74 (32.2%) nurses mentioned they received oral care training for ICU patients as part of their degree, whereas 40 (17.4%) of them received this training through additional courses, and another 42 (18.3%) of them were trained informally at the job. The remaining 74 (32.2%) reported not to have received any form of training; these findings are contrary to a study by Philip et al. [14], who reported 66% of their study population to have received training as part of their degree and only about 7.2% of them to have been trained at the job. Tabatabaei et al. [20] recognized in their study that most nurses do not receive adequate training as part of their curriculum and learn mostly informally about oral care for inpatients on the job.

Overall, the nurses responded favorably for the attitude set of questions; in general, most studies have reported the same regarding the attitude of nurses towards oral care of ICU patients (Alja'afreh et al. [21], Saddki et al. [22], and Philip et al. [14]). Though nurses have a favorable/positive attitude towards oral care, consistently there has been significant disagreement among nurses with regard to oral care being an unpleasant task and difficult to carry out (Kim et al. [23] and Narbutaitė et al. [24]), this was evident in the response of the present study population too, which could be attributed to factors such as, attention required towards other emergencies, anxiety of moving the intubation and difficulty in managing oral tissues and its bleeding tendencies, which could further complicate the care.

It was evident from the present study that nurses with higher experience (>10 years) at ICU feel the need for patients to be evaluated by dentists and help aid in drafting an oral care management strategy tailored based on patients' needs. Also, they are more likely, with experience, to believe that it's their responsibility to provide oral care to these patients. Contrastingly, higher experience does not ensure they are engaged in frequently providing oral care, as this aspect of increased frequency in providing oral care was noticed to be higher among the less experienced nurses (<5 years). These findings are in accordance with a study by Jun et al. on the role of dentists [25].

A very high number of nurses, 218 (94.8%), mentioned they provide an oral assessment to the ICU admitted patients within 24 hours. The response has been found to be high for this pertinent question in most studies from the past (Kelly et al. [26] and Philip et al. [14]). Just about 131 (57%) of the respondents mentioned that there exists a protocol for assessment and provision of oral care in the hospital. Contrastingly, 99 (43%) of them were unaware of the same. This finding is in line with most studies on the topic (Miranda et al. [27] and Jun et al. [25]).

Our study used the questionnaire after testing for its validity and reliability, which ensures the generalizability of our findings. Most developed countries do witness a mixed population representing various countries, so studying this variable is interesting and should be investigated, as well as comparison of curriculum related to oral health care emphasis is the need of the hour.

Limitations of the study

This study was carried out among nurses in various specialty hospitals in one medical city (King Abdulaziz Medical City, Riyadh). This questionnaire can be further put to use in investigating the knowledge, attitude, and practice of nurses in this region. Instead of convenient sampling as applied in this study, stratified cluster sampling can be used in future research, stratification of private and government hospitals based on clusters in the region. It was also observed in the study that the response rate was less, owing to their hectic schedule and multiple shifts, some nurses reported to various specialty ICUs, not merely only one specialty.

## Conclusions

Findings from the study can be used to generalize that the region has a highly diverse representation of nurses practicing at the ICU. As in most studies, this study also observed that most of the nurses practicing have a bachelor’s degree, whereas few possess a master’s degree. There were certain knowledge, attitude, and practice-based questions that elicited significant differences in response based on the qualification and experience of the nurses, likewise when the type of specialty in which they currently practice did not have any bearing on significance in response, this may be due to nurses practicing on rotation in different ICU settings, as and when posted. On the whole, nurses did possess fair knowledge, favorable attitudes, and reasonable practices while managing the oral health of ICU patients when analyzed alongside pertinent literature from various developed and developing countries around the world.
